# Pea Powdery Mildew and Pea Performance in Pea–Cereal Intercropping Under Temperate Continental Field Conditions: Yield, Seed Physical Quality, and Land-Use Efficiency Under Low Natural Disease Pressure

**DOI:** 10.3390/plants15101437

**Published:** 2026-05-08

**Authors:** Milosav Grčak, Dragan Grčak, Miroljub Aksić, Vera Rajičić, Slaviša Gudžić, Katerina Nikolić

**Affiliations:** 1 Faculty of Agriculture, University of Priština in Kosovska Mitrovica, Kopaonička bb, 38219 Lešak, Serbia; 2 Faculty of Agriculture, University of Niš, Kosančićeva 4, 37000 Kruševac, Serbia

**Keywords:** pea, intercropping, powdery mildew, yield, cereals, TSW, HLW, LER

## Abstract

Pea–cereal intercropping may combine ecological disease regulation with improved land-use efficiency, but field evidence for pea powdery mildew responses on the pea component under temperate continental conditions remains limited. A two-year field experiment (2017/2018 and 2018/2019) was conducted in Novi Sad, Serbia, to evaluate the effects of intercropping on pea powdery mildew disease index (DI%), pea grain yield, seed physical quality traits, and land-use efficiency. Winter pea cv. Kosmaj was grown as a sole crop or in mixed intercropping (70% pea + 30% cereal seeding rates) with wheat, triticale, rye, or oat in a randomized complete block design with four replicates. Powdery mildew DI% was assessed at BBCH 71–75, while pea grain yield, thousand-seed weight (TSW), hectoliter weight (HLW), and yield-based land equivalent ratio (LER) were determined at harvest. Under the low natural disease pressure recorded in the study, intercropping was associated with lower DI% than sole cropping (approximately 2.8-fold lower on seasonal means; *p* < 0.001), but DI% did not show a significant independent effect on pea grain yield, TSW, or HLW after accounting for year and cultivation system. Pea grain yield was generally lower under intercropping, although the magnitude of reduction depended on the cereal companion; pea–triticale maintained pea yield closest to the sole crop, whereas pea–oat showed the lowest pea yield. TSW and HLW were generally higher under intercropping, but additional analyses indicated that these traits reflected different response patterns. All intercrops achieved LER > 1, with the highest values recorded for pea–triticale.

## 1. Introduction

Conventional and simplified cropping systems often rely on repeated external inputs, including mineral fertilizers and chemical plant protection products, with well-documented concerns regarding soil quality, agroecosystem functioning, environmental sustainability, and public health [1,2,3,4,5]. Beyond these environmental concerns, simplified cropping systems may also increase disease pressure by maintaining continuous host availability and a relatively uniform canopy structure that can favor pathogen establishment and spread [6,7]. These limitations have strengthened interest in crop diversification strategies that support productivity while enhancing ecological regulation.

Intercropping, defined as the cultivation of two or more crop species in the same field during at least part of their growth cycle, has a long research tradition in temperate agriculture and has repeatedly been proposed as a means to improve land-use efficiency and overall productivity relative to monocultures [8,9,10,11]. More recently, intercropping has gained renewed attention as a strategy to improve resource-use efficiency, yield stability, and biological regulation within agroecosystems, particularly under climate variability and the need to reduce external inputs [12,13,14]. Among diversified systems, legume–cereal mixtures are of particular interest because functional complementarity between component species can improve the use of light, water, and nutrients compared with sole cropping [15,16,17]. As a result, such systems often achieve higher land-use efficiency, commonly quantified by the land equivalent ratio (LER), and may contribute to greater resilience under variable environmental conditions [13,18,19].

At the same time, the performance of intercropping systems is strongly context-dependent and shaped by interacting biophysical and management factors. Many studies have focused on individual outcomes, such as yield, rather than adopting a broader perspective that simultaneously considers productivity, crop health, and component-specific responses [20]. In addition, interspecific interactions may shift the competitive balance during the growing season, complicating cultivar choice and limiting the transferability of conclusions derived from pure stands [21]. Uncertainty regarding the relative contribution of each component at harvest remains a practical barrier to wider adoption of intercrops, particularly when the agronomic response of one component is prioritized over system-level performance alone [22].

Pea (*Pisum sativum* L.) is an important grain legume and a frequent component of cereal-based intercropping because of its capacity for biological nitrogen fixation and its agronomic compatibility with winter cereals. Pea–cereal systems have been reported to improve land-use efficiency and contribute to yield stability at the system level, while also offering opportunities to reduce external inputs [13,18]. Nevertheless, component-specific responses of winter pea under temperate continental conditions remain insufficiently characterized, particularly when multiple cereal companion species are evaluated within the same experimental framework.

Beyond yield-related outcomes, increased crop diversity may also contribute to plant disease management through several, often simultaneous, mechanisms. Differences in plant architecture, phenology, and growth dynamics can alter canopy structure and microclimate, thereby affecting pathogen dispersal and infection efficiency. Intercropping may reduce effective host density (“dilution effect”), create physical barriers to spore movement, and modify within-canopy humidity and temperature, all of which are potentially relevant for foliar disease development [23,24,25,26,27]. However, the magnitude and consistency of these effects depend on disease pressure, weather conditions, and mixture design, and therefore cannot be assumed to be uniform across pathosystems or growing environments.

Powdery mildew of pea, caused by Erysiphe pisi, is a major foliar disease that typically intensifies during later growth stages under warm conditions and elevated relative humidity. By reducing photosynthetically active leaf area, the disease can negatively affect both yield and seed quality [28,29,30,31,32]. Although disease-related research in cereal–legume intercrops has often emphasized pathogens of the cereal component, fewer field studies have explicitly quantified the response of pea powdery mildew on the pea component within pea–cereal intercropping systems, despite the epidemiological relevance of late-season mildew epidemics [33,34]. Moreover, when disease suppression is observed in intercrops, its agronomic consequences for the pea component do not necessarily follow a simple or uniform pattern, because disease responses may be partly decoupled from component yield formation and seed-quality traits under mixed cropping conditions.

Therefore, the objective of the present study was to evaluate the effects of pea–cereal intercropping on (1) pea powdery mildew disease index (DI%) on winter pea, (2) pea grain yield and seed physical quality traits (TSW and HLW), and (3) land-use efficiency expressed as LER, under field conditions in a temperate continental climate. In the present study, TSW was interpreted primarily as an indicator of individual seed mass and seed filling, whereas HLW reflected the bulk density of the harvested seed lot; these traits were therefore not assumed a priori to respond identically under intercropping. We hypothesized that, under the natural disease pressure observed in the study seasons, intercropping would be associated with lower DI% than sole cropping, while pea component yield and seed physical quality would vary among cereal companion species and between growing seasons.

## 2. Results

The general linear model showed that year and cultivation system significantly affected winter pea grain yield, seed physical quality traits (TSW and HLW), and powdery mildew disease index (DI%) (Table 1). Pea grain yield was significantly influenced by cultivation system (*p* < 0.001) and year (*p* = 0.002), whereas the year × cultivation system interaction was not significant (*p* = 0.194). Thousand-seed weight (TSW) and hectoliter weight (HLW) were likewise significantly affected by year (*p* = 0.005 and *p* < 0.001, respectively) and cultivation system (*p* < 0.001 and *p* = 0.002, respectively), with no significant year × cultivation system interaction for either trait (Table 1). In contrast, powdery mildew DI% was significantly affected by year (*p* < 0.001), cultivation system (*p* < 0.001), and their interaction (*p* = 0.001), indicating that the magnitude of DI% differences among cultivation systems varied between seasons.

When DI% was included as a covariate in separate ANCOVA/GLM models, it was not a significant predictor of pea yield (*p* = 0.221), TSW (*p* = 0.125), or HLW (*p* = 0.319) after accounting for year and cultivation system (Table 1). Accordingly, the reduction in DI% observed under intercropping should be interpreted as a treatment-associated disease response within the range of natural disease pressure recorded in the study, rather than as evidence that variation in DI% independently explained subsequent differences in pea yield or seed physical quality.

Across the two seasons, intercropping and sole cropping showed contrasting response patterns for the pea component (Table 2). Under the observed low natural disease pressure, intercropping was associated with lower pea DI% than sole cropping, whereas pea grain yield generally decreased under intercropping. By contrast, both TSW and HLW were higher in intercropped pea than in sole-cropped pea, although the magnitude of these responses varied among cereal companion species and between years (Table 2). At the system level, all intercrops achieved LER values greater than 1, indicating a land-use advantage relative to the corresponding sole crops, with the greatest advantage observed in the pea–triticale combination (Section 2.4).

### 2.1. Lower Pea Powdery Mildew DI% in Pea–Cereal Intercrops Under Observed Low Natural Disease Pressure

Powdery mildew disease index (DI%) on winter pea differed between the two growing seasons, indicating season-dependent variation in disease development. Mean DI% values were higher in 2019 than in 2018, consistent with the significant main effect of year (*p* < 0.001) (Table 1).

Across both seasons and within the range of natural disease pressure recorded in this study, DI% was consistently lower in pea grown in pea–cereal intercrops than in sole-cropped pea (Table 3). In sole-cropped pea, mean DI% reached 13.05% in 2018 and 14.87% in 2019, whereas all intercropping combinations showed lower values, ranging from 3.99% to 5.20% in 2018 and from 3.70% to 6.70% in 2019 (Table 3). Based on seasonal means, DI% decreased from 13.05% to 4.61% in 2018 and from 14.87% to 5.31% in 2019, corresponding to an approximately 2.8-fold lower disease index in intercropped than in sole-cropped pea in both seasons.

The magnitude of DI% differences between sole cropping and intercropping varied between years, as indicated by the significant year × cultivation system interaction (*p* = 0.001) (Table 1). Tukey’s HSD comparisons further showed differences among cereal companion crops within each season (Table 3). In 2019, the oat intercrop had the lowest mean DI% (3.70%), which was significantly lower than that of the wheat intercrop (6.70%), while the triticale and rye intercrops showed intermediate values. In 2018, all intercropping treatments reduced DI% relative to sole cropping, with the oat intercrop again showing the lowest mean DI% (3.99%), although differences among intercropping combinations were less pronounced than in 2019 (Table 3).

Overall, these results indicate that, under the observed untreated field conditions and relatively low natural disease pressure, pea–cereal intercropping was associated with lower powdery mildew DI% than sole cropping, while the extent of reduction depended on both season and cereal companion species.

### 2.2. Pea Grain Yield and Seed Physical Quality (TSW and HLW) Under Sole Cropping and Intercrops

Both year and cultivation system significantly affected pea grain yield and seed physical quality traits (TSW and HLW) (Table 1). Pea grain yield was significantly influenced by cultivation system (*p* < 0.001) and year (*p* = 0.002), whereas the Year × Cultivation system interaction was not significant (*p* = 0.194), indicating that the relative ranking of cultivation treatments for pea yield was broadly consistent across seasons. Likewise, TSW and HLW were significantly affected by both year (*p* = 0.005 and *p* < 0.001, respectively) and cultivation system (*p* < 0.001 and *p* = 0.002, respectively), while the Year × Cultivation system interaction was not significant for either trait (Table 1). Year-specific treatment means are shown in Table 2 for descriptive comparison, whereas pooled Tukey comparisons across years are presented in Table 4 because the Year × Cultivation system interaction was not significant for yield, TSW, and HLW.

Across treatments, mean pea yield was higher in 2019 than in 2018 (Table 2). When averaged across years, Tukey’s HSD comparisons showed that pea yield in the triticale intercrop did not differ from that in the sole crop (both group A), whereas yields were significantly lower in the wheat intercrop (group B) and lowest in the oat intercrop (group C), with the rye intercrop showing intermediate performance (group BC) (Table 4).

Seed physical quality traits showed a contrasting response pattern relative to pea yield. Across treatments, mean TSW was higher in 2018 than in 2019 (Table 2). Pooled Tukey comparisons showed that all intercropping combinations had significantly higher TSW than the sole crop (all intercrops in group A vs. sole crop in group B), whereas differences among cereal companion species were not significant for this trait (Table 4). For HLW, mean values were higher in 2019 than in 2018 (Table 2). Pooled comparisons further indicated that wheat, triticale, and oat intercrops formed the highest HLW group (A), rye showed intermediate values (AB), and the sole crop had the lowest HLW (B) (Table 4).

The treatment means in Table 2 further illustrate that the response of the pea component differed among cereal companions. In both seasons, sole-cropped pea had the highest or among the highest pea grain yields, whereas intercropping generally reduced pea component yield. At the same time, all intercrops showed higher TSW than the sole crop in both years, while HLW also tended to be higher under intercropping, although the magnitude of HLW increase varied more among cereal companions and between seasons than the TSW response (Table 2).

Overall, the univariate analyses showed a consistent contrast between pea component yield and seed physical quality under intercropping: pea grain yield generally decreased, whereas TSW and HLW increased relative to sole cropping. Additional relationships among pea yield components, pea yield, and seed physical quality traits are presented separately in Section 2.3.

### 2.3. Associations of Pea Yield Components with Pea Yield and Seed Physical Quality Traits (TSW and HLW)

To further examine why TSW and HLW did not respond identically across cultivation treatments, additional exploratory analyses were performed using plot-level observations. Because TSW and HLW were determined at the plot level, plant-based morphological and yield-component traits were first expressed as plot means and then analyzed together with pea grain yield, powdery mildew disease index (DI%), TSW, and HLW. Pairwise relationships among traits were summarized using a correlation heat map, and a focused principal component analysis (PCA) was used to provide a multivariate summary of the main associations contributing to the contrasting behavior of TSW and HLW.

**Figure 1:**
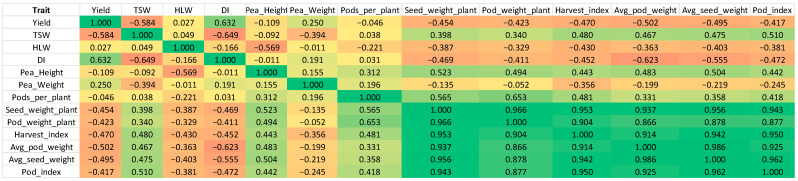
Correlation heat map showing plot-level associations among pea grain yield, powdery mildew disease index (DI%), seed physical quality traits (TSW and HLW), and selected pea morphological and yield-component traits. Correlation coefficients are shown within cells; positive and negative associations are represented by contrasting color gradients. Trait values used in the analysis were expressed at the plot level, with plant-based traits averaged within each replicate before analysis.

Plot-level correlation analysis showed that TSW and HLW were only weakly associated with each other (r = 0.049), indicating that the two seed physical quality traits did not capture the same dimension of variation (Figure 1). Pea grain yield was negatively associated with TSW (r = −0.584) but showed essentially no relationship with HLW (r = 0.027). Likewise, DI% was more strongly associated with TSW (r = −0.649) than with HLW (r = −0.166). TSW showed positive associations with several traits related to seed filling and reproductive allocation, including average seed weight (r = 0.475), harvest index (r = 0.480), and pod index (r = 0.510). In contrast, HLW showed a different pattern, with the strongest association observed for pea height (r = −0.569), while negative associations were also observed with average seed weight (r = −0.403), harvest index (r = −0.430), and pod index (r = −0.381). Overall, the correlation structure indicated that TSW was more closely aligned with traits describing individual seed filling and reproductive partitioning, whereas HLW followed a distinct association pattern.

**Figure 2:**
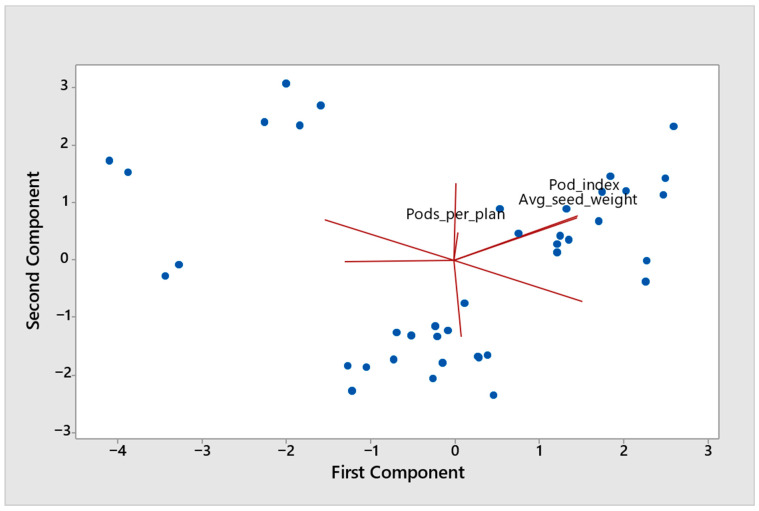
Principal component analysis (PCA) biplot based on plot-level values of pea grain yield, powdery mildew disease index (DI%), seed physical quality traits (TSW and HLW), and selected pea morphological and yield-component traits. Variables were standardized prior to analysis. Arrows represent variable loadings and their directions of association with the principal components, whereas points represent plot-level observations. The first two principal components summarize the main multivariate structure of trait relationships under sole cropping and intercropping conditions.

To provide a multivariate summary of these relationships, a focused PCA was performed using yield, TSW, HLW, DI%, pea height, pods per plant, average seed weight, and pod index (Figure 2). The first two principal components explained 71.2% of the total variation (PC1 = 40.8%, PC2 = 30.4%), indicating that most of the multivariate structure was captured in two dimensions. PC1 was primarily associated with TSW, average seed weight, and pod index, and opposed to DI% and pea grain yield. By contrast, HLW contributed little to PC1 and was mainly associated with PC2, together with pea height, but in the opposite direction. Pods per plant contributed predominantly to PC3 rather than to the first two components. The PCA therefore confirmed that TSW and HLW were not aligned along the same major axis of variation: TSW clustered more closely with seed-filling-related traits, whereas HLW was associated with a separate dimension represented primarily by pea height.

Based on the correlation and PCA patterns, additional regression analyses were used as complementary exploratory tools to quantify the strongest unadjusted plot-level associations for TSW and HLW. In these exploratory analyses, TSW showed its strongest negative association with DI%, whereas HLW showed its strongest association with pea height (Figure S1). These patterns further supported the view that TSW and HLW were linked to different sets of plot-level traits and should not be interpreted as interchangeable indicators of pea seed physical quality. However, these exploratory associations should be interpreted alongside the ANCOVA/GLM results, in which DI% was not retained as an independent predictor of TSW or HLW after accounting for year and cultivation system.

Taken together, the correlation heat map, focused PCA, and complementary exploratory regression analyses consistently showed that TSW and HLW were only weakly associated with each other and were linked to different trait complexes. TSW was more closely associated with DI% and traits related to seed filling and reproductive allocation, whereas HLW was more strongly associated with pea height. These results indicate that the increase in seed physical quality observed under intercropping was not expressed through a single uniform response pattern, but rather through distinct relationships for TSW and HLW.

### 2.4. Land-Use Efficiency of Pea–Cereal Intercropping Systems (LER)

**Figure 3:**
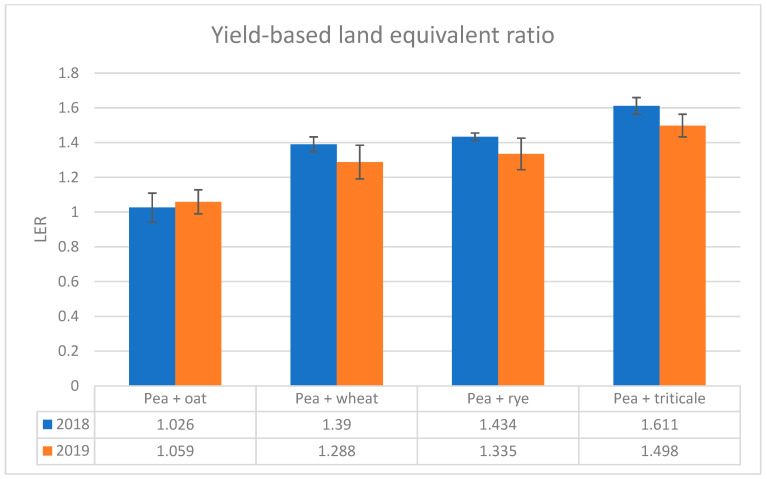
Yield-based land equivalent ratio (LER) for winter pea intercropped with wheat, rye, triticale, and oat during the 2018 and 2019 seasons. Values are means ± SE (*n* = 4). LER values greater than 1 indicate a land-use advantage of intercropping relative to the corresponding sole crops.

To complement the pea component responses presented in Section 2.2 and Section 2.3, system-level performance was evaluated using yield-based land equivalent ratio (LER). In both growing seasons, all pea–cereal intercrops achieved LER values greater than 1, indicating a land-use advantage over the corresponding sole crops (Figure 3).

In 2018, LER ranged from 1.026 in the pea–oat intercrop to 1.611 in the pea–triticale intercrop. In 2019, it ranged from 1.059 (pea–oat) to 1.498 (pea–triticale). Across both seasons, pea–triticale showed the highest LER, followed by pea–rye and pea–wheat, whereas pea–oat showed only a marginal advantage over sole cropping. Although LER values for wheat, rye, and triticale intercrops were lower in 2019 than in 2018, the overall ranking of intercrop combinations remained similar between years (Figure 3).

Partial LER values further showed that total LER reflected the combined contributions of both pea and cereal components, and that these contributions varied among intercrop combinations and between years (Table S1). Across seasons, pLER ranged from 0.458 to 1.027 in 2018 and from 0.516 to 1.000 in 2019, whereas cLER ranged from 0.568 to 0.649 in 2018 and from 0.499 to 0.742 in 2019.

Absolute system productivity, expressed as total intercrop yield (pea + cereal component yield), showed that intercrop combinations with higher LER did not necessarily have the highest total intercrop yield (Table 5). In both seasons, the ranking based on total intercrop yield was not identical to the ranking based on LER. In particular, pea–triticale showed the highest LER, whereas pea–rye achieved the highest absolute total intercrop yield, confirming that relative land-use efficiency and absolute system productivity represented complementary rather than interchangeable performance criteria in the present dataset.

## 3. Discussion

Intercropping research has frequently emphasized system productivity, whereas disease responses of the pea component have been quantified less often than those of the cereal companion. The present two-year field study should therefore be interpreted primarily as an intercropping study conducted under low natural powdery mildew pressure, in which disease intensity, pea component performance, seed physical quality, and system-level land-use efficiency responded in partially decoupled ways. Under these conditions, cereal companion identity emerged as the main organizing factor for pea component performance, while the observed reduction in DI% represented an important but not independently yield-determining part of the overall intercrop response.

### 3.1. Pea Powdery Mildew Responses Under Low Natural Disease Pressure

Pea–cereal intercropping was associated with a lower pea powdery mildew disease index (DI%) than sole cropping in both growing seasons. However, this result should be interpreted within the range of natural disease pressure recorded in the present study, because disease levels remained relatively low in both years, and DI% was assessed once at the late reproductive stage. Under these conditions, the observed reduction in DI% is best interpreted as evidence of a treatment-associated epidemiological response rather than as a basis for broad conclusions about disease control effectiveness under all field conditions [26,34].

The direction of the response agrees with previous work showing that intercropping frequently reduces foliar disease severity relative to monocropping through a combination of reduced effective host density, physical interference with pathogen dispersal, and intercrop-induced changes in canopy structure and microclimate [26,35,36,37,38]. Pea-specific evidence also supports such a pattern, although the magnitude of disease suppression depends strongly on the companion species and cropping design. Villegas-Fernández et al. [34] reported that pea intercropping reduced powdery mildew when pea was grown with barley or faba bean, whereas the effect was weaker or absent with wheat, and they identified the barrier effect of the companion crop as a major explanatory mechanism. Similarly, Živanov et al. [33] found that pea–oat intercropping reduced several disease parameters relative to pea monoculture, although powdery mildew reduction at the whole-plant level was less clear-cut than for other measured disease traits. Together, these studies support the view that the lower DI% recorded in our intercrops is biologically plausible, but also that companion-species effects should not be treated as uniform [26,33,34].

The companion-species ranking observed here also provides a cautious link between the disease data and likely intercrop architecture. In the present dataset, the oat intercrop was associated with the lowest DI%, whereas wheat tended to show the highest DI% among intercrops. A biologically plausible interpretation is that different cereal companions modified the pea canopy environment to different degrees through differences in plant architecture, host dilution, and partial physical disruption of pathogen spread. However, because within-canopy humidity, airflow, leaf wetness, and light interception were not measured directly, these mechanisms should be regarded as plausible explanatory pathways rather than as experimentally demonstrated causes in the present study.

At a broader mechanistic level, recent reviews emphasize that plant responses to biotic and environmental stress are shaped not only by whole-canopy conditions but also by redox-mediated signaling and by spatially heterogeneous, cell-type-specific responses within tissues. Reactive oxygen and nitrogen species (ROS/RNS) are increasingly recognized as central components of plant defense signaling networks, while emerging cell-type-resolved approaches show that stress responses are not deployed uniformly across plant tissues. Although such processes were not measured in the present field experiment, these recent perspectives support the view that intercrop-mediated disease responses are likely to arise from spatially heterogeneous plant–pathogen–environment interactions rather than from a single uniform mechanism [39,40].

The significant Year × Cultivation interaction for DI% indicates that the magnitude of disease reduction varied between seasons, even though the direction of the intercrop effect remained consistent. This is also consistent with the epidemiology of intercropping-mediated disease regulation, which is known to depend on the interaction among host arrangement, canopy development, and season-specific environmental conditions [26,34]. In our dataset, disease pressure was somewhat higher in 2019 than in 2018, and differences among cereal companions were also more evident in 2019. This year-to-year difference is consistent with the contrasting seasonal distribution of rainfall and early spring temperatures observed between the two study seasons relative to the long-term climatic context. The oat intercrop showed the lowest DI%, whereas wheat tended to show the highest DI% among intercrops, suggesting that the companion species influenced disease development differently under the prevailing field conditions.

A critical point, however, is that the lower DI% observed under intercropping did not translate into a significant independent effect of DI% on pea grain yield, TSW, or HLW once year and cultivation system were accounted for in the ANCOVA/GLM analyses. This does not mean that powdery mildew is agronomically unimportant in pea. On the contrary, pea powdery mildew is well known to reduce yield and yield-related traits under conducive conditions, and substantial losses have been reported in the literature when epidemics are more severe or prolonged [41,42]. Rather, our results indicate that, within the relatively narrow and low disease range recorded here, the experiment did not capture a sufficiently strong disease gradient to resolve a clear independent contribution of DI% to pea yield or seed physical quality. Therefore, the absence of a significant adjusted DI% effect in our models should be interpreted as a limitation of the observed disease scenario, not as evidence that powdery mildew has no effect on pea productivity. This caution also applies to cultivar interpretation: because only one pea cultivar was included and no independent resistance screening was performed, the present dataset does not allow Kosmaj to be classified as resistant or tolerant to powdery mildew on the basis of the observed field response alone.

Overall, the present results support the conclusion that pea–cereal intercropping can reduce pea powdery mildew DI% under low natural disease pressure, but they also show that the agronomic implications of this reduction cannot be inferred directly from DI% alone. Stronger inference about the disease-management value of these systems will require experiments covering wider infection gradients, including controlled or artificially increased disease pressure, repeated disease assessments through time, and direct evaluation of companion-species effects on canopy traits and within-canopy conditions [26,34].

### 3.2. Companion-Species Effects on Pea Yield Performance

At the pea-component level, yield responses to intercropping were clearly companion-species-dependent. Although pea grain yield was generally lower in intercrops than in the sole crop, the magnitude of reduction was not uniform across cereal companions. The triticale intercrop maintained pea yield at a level comparable to the sole crop in the pooled comparison, whereas wheat and rye reduced pea yield, and the oat intercrop showed the lowest pea yield. This pattern indicates that cereal identity strongly influenced the balance between complementarity and competition in the present experiment.

Such a response is consistent with the broader intercropping literature, in which cereal–legume mixtures frequently improve system-level performance while reducing the legume component yield because cereals are often more competitive for light, water, and soil mineral nitrogen [15,16,21,43]. In cereal–pea intercrops, this competitive imbalance is especially important when the cereal component develops rapidly and increasingly dominates the canopy, thereby constraining the pea component even when total intercrop productivity remains favorable [15,16,21,43]. Therefore, the lower pea yields recorded in several intercrop combinations in our study should not be interpreted as anomalous, but rather as a common component-level response in cereal–legume mixtures.

At the same time, our results show that this general tendency cannot be interpreted uniformly across cereal companions. The pea–triticale combination was the most favorable for pea yield preservation in the present dataset. This agrees with previous work showing that winter pea intercropped with triticale can maintain substantial pea seed yields, even when intercrop pea yield remains somewhat below that of the sole crop, and that the success of winter pea intercrops depends strongly on the specific cereal companion and the growing conditions [44,45]. In this context, the comparatively good performance of pea with triticale in our study suggests a more favorable competitive balance than in the wheat-, rye-, or oat-based mixtures, rather than a general intercrop effect independent of companion identity.

Although belowground processes were not measured directly in the present study, the species-dependent yield penalties observed here are consistent with differences in root-mediated competition and complementarity among cereal companions. In cereal–legume intercrops, variation in nitrogen acquisition, possible facilitative effects linked to biological N fixation, and competition for soil water may all contribute to how strongly the cereal companion constrains or preserves the pea component. From a practical standpoint, this also means that intercrop optimization should not focus on disease suppression alone. Instead, companion choice, cultivar combination, and sowing ratio should be considered together. This is especially relevant for pea–oat, where the lowest DI% coincided with the strongest pea yield penalty, suggesting that lower cereal dominance or alternative cultivar combinations may be needed to retain disease-related benefits without excessive loss of pea component yield [15,16,21,43,44,45,46,47].

The oat intercrop provides the clearest example that lower disease intensity did not automatically translate into better pea yield performance. In our study, oat was associated with the lowest DI% but also with the lowest pea grain yield. This indicates that disease suppression and yield preservation were at least partly decoupled under the observed field conditions. A likely explanation is that the oat companion exerted stronger competitive pressure on the pea component than the other cereals, thereby overriding any potential yield benefit associated with lower DI%. This interpretation is supported by previous studies showing that oat can behave as a dominant partner in oat–pea intercrops and that pea–oat intercropping may significantly reduce pea grain yield, depending on sowing ratio, nitrogen regime, and site-year conditions [46,47].

The wheat and rye intercrops showed intermediate pea yield responses, again indicating that the effect of intercropping on pea yield was species-dependent rather than uniform across cereals. Published studies on cereal–pea mixtures likewise show that companion choice and cultivar combination are critical determinants of pea performance, and that not all cereal companions impose the same competitive cost on the legume component [44,45]. Thus, the present results support the view that companion-species identity must be considered explicitly when interpreting pea yield responses in intercropping systems.

A critical implication of these findings is that the lower DI% observed in intercrops should not be used to explain pea yield responses directly. As shown by the ANCOVA/GLM analyses, DI% was not a significant independent predictor of pea yield after year and cultivation systems were taken into account. Therefore, the yield differences among intercrop combinations in our study are better interpreted primarily in terms of companion-species effects on pea performance than as direct consequences of powdery mildew reduction. Given the relatively low disease pressure recorded in both years, the present dataset does not support a simple disease-driven explanation for the observed pea yield pattern.

Overall, the yield results indicate that intercropping did not affect pea performance in a uniform way. Instead, cereal companion species modified the pea response differently, with triticale providing the most balanced outcome for pea yield, oat imposing the strongest yield penalty, and wheat and rye showing intermediate effects. Because the mixtures were established according to agronomically recommended seeding-rate proportions rather than as an iso-density replacement design, these conclusions should be interpreted as applying to practical mixed-intercrop systems and not as isolating density-normalized species effects. This species-dependent pattern is central to the interpretation of pea performance in the present study and should be considered together with the seed-quality and system-level results discussed in the following sections.

### 3.3. Divergent Responses of TSW and HLW and Their Trait Associations

Although both TSW and HLW were generally higher under intercropping than under sole cropping, the exploratory correlation heat map, focused PCA, and complementary regression analyses consistently indicated that these two traits did not behave as interchangeable indicators of pea seed physical quality. In practical terms, TSW captured variation more closely related to individual seed mass and seed filling, whereas HLW reflected the bulk-density-related behavior of the harvested seed lot. Thus, the increase in seed physical quality observed under intercropping was not expressed through a single uniform response, but through at least two partially distinct dimensions of pea seed performance.

TSW was more closely associated with average seed weight, pod index, and, in the simple plot-level regressions, with DI%. This pattern is biologically plausible because thousand-seed weight primarily reflects the mass of individual seeds and is therefore closely related to seed filling during the reproductive phase [48]. In pea, seed weight and related yield components are known to respond to conditions that alter assimilate supply, partitioning, and reproductive development, including source–sink balance and crop stand structure [48,49,50]. The positive associations of TSW with average seed weight and pod index in our dataset are therefore consistent with the interpretation that TSW captured a seed-filling-related dimension of intercrop response.

By contrast, HLW followed a different pattern. In our analyses, HLW was only weakly related to TSW and was more strongly associated with pea height than with average seed weight or pod index. This distinction is consistent with the physical meaning of hectoliter weight itself. Unlike TSW, which reflects the mass of individual seeds, HLW reflects the mass of a seed lot within a given volume and therefore depends on the bulk physical properties of the harvested seed fraction. In a pea-specific physical properties study, Yalçın et al. [51] showed that as moisture content increased, thousand-seed mass increased, whereas bulk density decreased, demonstrating directly that seed mass and bulk-density-related properties may diverge even in pea, whose seeds are relatively rounded. A similar separation between these traits was observed in a pea-based intercropping study by Jaskulska et al. [52], where the weight of a thousand pea seeds differed significantly among cropping variants, whereas the weight of a hectoliter of pea seeds did not. Taken together, these findings support the interpretation that HLW and TSW should not be expected to respond identically, even within the same crop. Although demonstrated here with pea-specific evidence, this distinction is consistent with the broader grain-quality literature, in which test weight/specific weight is likewise interpreted as a bulk-density-related property influenced not only by individual grain mass but also by grain density and packing efficiency, indicating that the physical basis of HLW is not unique to cereals but reflects a more general seed-lot property [53,54,55].

This distinction also helps explain why the relationship with pea grain yield was stronger for TSW than for HLW. In our dataset, pea grain yield was negatively associated with TSW, whereas its association with HLW was negligible. This suggests that the trade-off between pea component yield and seed physical quality was expressed more strongly through individual seed mass than through hectoliter weight. Similar patterns have been reported in other field pea intercrop studies. Bailey-Elkin et al. [56], for example, found that intercrops reduced field pea grain yield but increased field pea seed mass, indicating that lower pea yield may coincide with heavier seeds rather than with parallel changes in all quality traits. In this context, our results suggest that the reduced pea yield observed in some intercrop combinations was accompanied by improved individual seed filling, whereas HLW reflected a different bulk physical property of the seed lot.

The additional analyses also clarified how these quality responses related to powdery mildew DI%. In simple regressions, DI% was strongly associated with TSW but not with HLW. However, in the ANCOVA/GLM analyses, DI% was not a significant independent predictor of either TSW or HLW once year and cultivation system were taken into account. Therefore, the strong unadjusted association between DI% and TSW should be interpreted as part of the broader treatment structure rather than as evidence that powdery mildew independently determined seed physical quality in the present experiment. Under the relatively low disease pressure recorded in both years, the increase in TSW is better interpreted as part of the overall intercrop response than as a direct consequence of lower DI%.

Overall, the present results show that seed physical quality responses of pea under intercropping were trait-specific rather than uniform. TSW was more closely associated with seed-filling-related traits and with the unadjusted disease gradient, whereas HLW was associated with a different structural dimension represented mainly by pea height. This distinction is important because it shows that increases in TSW and HLW under intercropping should not automatically be treated as equivalent or assumed to arise from the same biological processes.

### 3.4. Land-Use Efficiency in Relation to Pea Component Responses

Yield-based land equivalent ratio (LER) is one of the most widely used indices for quantifying the land-use advantage of intercropping relative to sole cropping, but its interpretation depends on how component yields are standardized against appropriate sole-crop references and on how component contributions are reported [57,58].

In the present study, all pea–cereal intercrops had LER values greater than 1 in both growing seasons, indicating a consistent land-use advantage under temperate continental field conditions. The highest LER values were recorded for pea–triticale in both years, followed by pea–rye and pea–wheat, whereas pea–oat showed only a marginal advantage over sole cropping. This ranking is consistent with our component-level results, in which pea–triticale also showed the most balanced outcome for the pea component, while pea–oat combined the lowest pea yield with only a slight system-level advantage.

The magnitude of the land-use advantage observed here, particularly for pea–triticale, is higher than the global mean values typically reported for grain intercrops in meta-analyses, while still remaining within the range documented across different species combinations and production contexts [59,60]. Independent evidence also supports triticale as a high-performing companion for winter pea. Klimek-Kopyra et al. [44] reported the highest total LER for winter pea intercropped with triticale, while pea seed yield in those mixtures remained agronomically high, although lower than in the corresponding sole crops. Together with our results, this supports the interpretation that triticale can provide a favorable balance between system-level efficiency and pea–component performance.

At the same time, LER should not be over-interpreted as a single-number ranking of the “best” intercrop. Partial LER values (pLER for pea and cLER for cereals) are important because they clarify whether total LER is driven mainly by the cereal component, the pea component, or both [57,59]. In our dataset, pLER and cLER varied among intercrop combinations and between years, confirming that total LER reflected different balances of component contributions rather than a uniform mechanism of land-use advantage.

A further important point is that LER expresses relative land-use efficiency and does not by itself indicate absolute productivity. For this reason, LER should be interpreted together with absolute intercrop yields and component yields [61]. This distinction was evident in our study: pea–triticale showed the highest LER, whereas pea–rye produced higher total intercrop yield in both seasons. Thus, the intercrop combination that performed best in terms of relative land-use efficiency was not identical to the one with the highest absolute total yield.

Overall, the LER results support the conclusion that pea–cereal intercropping improved system-level land-use efficiency in both years, but they also show that intercrop ranking depended on the performance criterion considered. In the present study, pea–triticale emerged as the most balanced combination when both pea-component responses and system-level efficiency were taken into account, whereas pea–oat provided only a limited system-level advantage despite its lower pea disease index.

## 4. Materials and Methods

The field experiment was conducted over two growing seasons (2017/2018 and 2018/2019) at the experimental field of the Institute of Field and Vegetable Crops, Novi Sad, Serbia, located at Rimski Šančevi (45°19′ N, 19°50′ E; 80 m a.s.l.). Winter pea cv. Kosmaj was used as the legume component because it is a locally adapted and agronomically relevant winter pea cultivar developed and officially released by the Institute of Field and Vegetable Crops, Novi Sad. However, the present study was not designed to test cultivar resistance to powdery mildew independently, and the observed low disease pressure should not be interpreted as evidence that Kosmaj is inherently resistant or tolerant. Pea was intercropped with winter wheat (Ilina), winter triticale (Odisej), winter rye (Savo), and winter oat (Jadar). All cultivars used in the study were developed and officially released by the Institute of Field and Vegetable Crops, Novi Sad. Winter crops were sown at the optimal time in October in both growing seasons, in accordance with regional agronomic recommendations. The soil at the experimental site was a slightly calcareous loamy chernozem. Prior to trial establishment (before the first growing season), the topsoil was analyzed and had the following properties: pH(KCl) 7.43, pH(H_2_O) 8.08, CaCO_3_ 4.61%, humus 2.85%, total N 0.21%, available P (Al-P_2_O_5_) 17.4 mg 100 g^−1^ soil, and available K (Al-K_2_O) 30.5 mg 100 g^−1^ soil.

Soil preparation before sowing included ploughing, followed by disc harrowing and cultivation. Basal fertilization was applied in October before winter sowing using monoammonium phosphate (MAP, 12:52:0) at a rate of 200 kg ha^−1^, corresponding to 24 kg N ha^−1^ and 104 kg P_2_O_5_ ha^−1^. Potassium fertilization was not applied because the pre-sowing soil analysis indicated a high level of available K (Al-K_2_O: 30.5 mg 100 g^−1^ soil), and K was therefore not considered limiting for crop establishment under the conditions of the experiment. No additional mineral fertilization was applied in spring. No commercial Rhizobium/Bradyrhizobium inoculant was applied before sowing. No fungicides were applied during the study, to allow assessment of the effect of pea–cereal intercropping on powdery mildew development in the absence of chemical disease control.

A randomized complete block design (RCBD) with four replicates was used. The plot area was 5 m^2^ per replicate. The study employed a mixed intercropping system, in which pea and cereal species were grown simultaneously within the same plot area without a defined row alternation pattern. Pea and cereal components were sown sequentially in two passes: pea was sown first at a depth of 4–5 cm, followed by the cereal component at a depth of 3–4 cm to accommodate crop-specific germination requirements.

Seeding rates in intercropping were adjusted according to the planned mixture proportions, with pea sown at 70% and cereals at 30% of their respective recommended sole-crop seeding rates. The recommended seeding rate for winter pea cultivar Kosmaj in sole cropping is 140 kg ha^−1^. In conventional sole cropping, the recommended seeding rates for the cereal cultivars are 220 kg ha^−1^ for wheat (Ilina), 275 kg ha^−1^ for triticale (Odisej), 153 kg ha^−1^ for rye (Savo), and 158 kg ha^−1^ for oat (Jadar). Expressed as target germinable seeds m^−2^, the intercrop design corresponded approximately to 103.6 for pea, 159.0 for wheat, 150.0 for triticale, 150.0 for rye, and 135.0 for oat. The intercrops were thus established as agronomically realistic mixed intercrops based on recommended sole-crop rates rather than as an iso-density replacement design. To improve transparency regarding stand establishment, post-winter established plant density was determined in late March in both seasons; these data are presented in the Supplementary Material (Table S2).

### 4.1. Disease Assessments

Powdery mildew was assessed at the pod development stage (BBCH 71–75), when pods were fully formed, a phenological phase conducive to the development of pea powdery mildew [62,63]. Ten plants were randomly selected from each replicate, resulting in a total of 40 assessed plants per crop type and cultivation system. On each plant, powdery mildew severity was visually estimated as the proportion of leaf area showing disease symptoms.

Disease severity was scored on an ordinal 0–9 scale adapted from Saari and Prescott [64], where 0 indicates the absence of observable symptoms and 9 indicates ≥95% of the leaf area exhibiting symptoms (Table 6). Similar severity scales have been applied in pea powdery mildew studies by Warkentin et al. [65] and Falloon et al. [66]. Severity scores were subsequently converted to a disease index (DI%) to express overall disease intensity at the plot level based on the frequency distribution of the 0–9 ratings.

The disease index (DI%) was calculated to quantify plot-level disease intensity from the distribution of 0–9 severity scores according to the Townsend–Heuberger approach [67], using the following equation (Equation (1)):

(1) DI(%)=(∑(v×n))/(i×N)×100

where v is the numerical severity score (0–9), n is the number of plants receiving a given score, i is the maximum score (9), and N is the total number of assessed plants. DI% provides a normalized estimate of overall disease intensity, reflecting both the distribution of severity ratings and the severity of symptoms, and facilitates comparisons among cultivation treatments and growing seasons.

### 4.2. Harvest, Yield, Seed Parameters, and Selected Pea Traits

Pea was harvested at the beginning of July at full physiological maturity. Harvest timing was synchronized across treatments to ensure comparability between sole-cropped and intercropped pea. In intercropping treatments, pea was harvested separately from the cereal component because the higher threshing drum speed required for cereals may cause mechanical damage to pea seeds. Therefore, pea plants were manually pulled from the plots before cereal harvest, collected in sacks, and subsequently threshed using a combine harvester. Because the plots were small and harvest was performed carefully for each replicate, pod and seed losses during manual pulling were considered minimal; however, they were not quantified separately and should therefore be regarded as a methodological limitation. Pea grain yield was expressed at 15% moisture content. Cereal components were harvested subsequently by a combine harvester at full grain maturity (harvest maturity), and cereal grain yield was also recorded at 15% moisture content. Grain quality traits, including thousand-seed weight (TSW) and hectoliter weight (HLW), were determined from plot-level grain samples after harvest.

To support the association analyses presented in Section 2.3, additional pea morphological and yield-component traits were measured at harvest. In each replicate plot, 10 pea plants were randomly sampled and analyzed individually. Pea height (cm), pea weight (g plant^−1^), pods per plant, seeds per pod, seed weight per plant (g plant^−1^), and pod weight per plant (g plant^−1^) were recorded. From these data, harvest index (%), average pod weight (g), average seed weight (g), and pod index (%) were calculated. Harvest index was calculated as seed weight per plant/pea weight per plant × 100, average pod weight as pod weight per plant/pods per plant, average seed weight as seed weight per plant/pods per plant, and pod index as seed weight per plant/pod weight per plant × 100. For association analyses, all plant-based traits were averaged within each replicate to obtain a single plot-level value, matching the experimental-unit level used for yield, DI%, TSW, and HLW.

### 4.3. Land Equivalent Ratio (LER)

The land equivalent ratio (LER), calculated on a yield basis, was used to assess land-use efficiency in intercropping systems relative to sole cropping. It represents the area required under sole cropping to produce the same combined yield as a unit area of intercropping [58]. LER values greater than 1 indicate a land-use advantage of intercropping, whereas values lower than 1 indicate a disadvantage. For example, an LER of 1.3 indicates that sole cropping would require 30% more land to achieve the same total yield as one hectare of an intercropped system.

Yield-based LER was calculated as the sum of the partial LER values of the cereal and pea components according to the standard formulation of Mead & Willey [58] (Equation (2)):

(2) $$LER\;yield=\frac{YIELD\;Cereal(intercrop)}{YIELD\;Cereal(sole\;crop)}+\frac{YIELD\;Pea(intercrop)}{YIELD\;Pea\;(sole\;crop)}$$

### 4.4. Climatic Conditions During the Experimental Period

**Figure 4:**
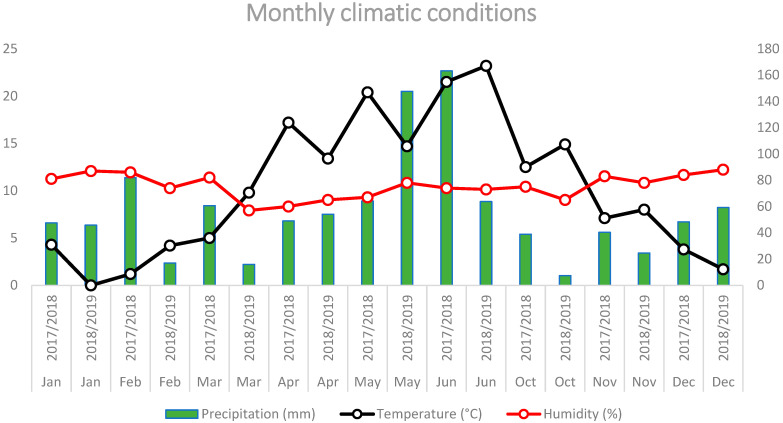
Monthly climatic conditions during the two growing seasons relevant to the field experiment (2017/2018 and 2018/2019): mean air temperature (°C), relative air humidity (%), and precipitation (mm). Only the months corresponding to the crop-growing period and field observations are shown. Meteorological data were obtained from the Republic Hydrometeorological Service of Serbia (RHMZ).

Climatic conditions differed between the two growing seasons (2017/2018 and 2018/2019). Meteorological data for the experimental seasons, including mean monthly air temperature, relative air humidity, and monthly precipitation, were obtained from the Republic Hydrometeorological Service of Serbia (RHMZ) and are presented in Figure 4. Only the months relevant to the crop-growing period and field observations are shown in the figure. For a broader climatic context, long-term monthly normals (1991–2020) from the Rimski Šančevi meteorological station (45°20′ N, 19°51′ E; 84 m a.s.l.) were used as a reference for comparison with the two experimental growing seasons (Table S3).

Compared with the long-term reference, the two study seasons differed in the seasonal distribution of precipitation and in early spring temperature patterns. The 2017/2018 season was characterized by higher precipitation during late spring and early summer, with a pronounced peak in June, whereas the 2018/2019 season showed lower precipitation during the autumn sowing period and early spring, followed by markedly increased rainfall in May. Temperature patterns also differed between seasons, with a warmer March but a cooler April in 2018/2019 compared with 2017/2018. Relative air humidity remained high in both seasons during late spring and early summer, i.e., during periods relevant to powdery mildew development.

For simplicity, the 2017/2018 and 2018/2019 growing seasons are referred to hereafter as 2018 and 2019 throughout the manuscript.

### 4.5. Statistical Methods

All univariate analyses were performed in Minitab 17 using plot-level data from a randomized complete block design with four replicates per treatment in each growing season. Pea grain yield, TSW, HLW, and powdery mildew disease index (DI%) were analyzed by general linear model (GLM) with Year, Cultivation treatment, and their interaction (Year × Cultivation treatment) included as fixed effects, while block (replicate) was included as a blocking factor. Statistical significance was assessed at α = 0.05. When overall effects were significant, means were separated using Tukey’s honestly significant difference (HSD) test at the 95% confidence level. Because the Year × Cultivation interaction was significant for DI%, Tukey comparisons for DI% were interpreted within each year; because this interaction was not significant for yield, TSW, and HLW, comparisons for these traits were presented as pooled across years. To assess whether variation in DI% contributed to variation in pea yield and seed physical quality, DI% was included as a covariate in separate ANCOVA/GLM models for yield, TSW, and HLW. Land equivalent ratio (LER), partial LER values, total intercrop yield, and component yield shares were calculated and presented as descriptive system-level indicators using mean ± SE (*n* = 4), whereas formal inferential comparisons were applied to pea-component traits through the GLM, ANCOVA/GLM, and Tukey’s HSD analyses described above.

Additional association analyses were performed to examine relationships among pea yield, DI%, TSW, HLW, and selected pea morphological and yield-component traits. Because TSW and HLW were measured once per plot, whereas plant-based traits were measured on 10 plants per plot, plant-based measurements were first averaged within each replicate and then analyzed as plot-level variables. Pairwise associations among traits were summarized using Spearman’s rank correlation coefficient (rho) and visualized as a correlation heat map. Principal component analysis (PCA) was used as an exploratory multivariate method to summarize trait associations; variables were standardized prior to PCA. An overview PCA was performed using pea yield, TSW, HLW, and DI% (Figure S2), and a focused PCA was performed using pea yield, DI%, TSW, HLW, pea height, pods per plant, average seed weight, and pod index.

To quantify the strongest plot-level associations identified by the correlation and PCA analyses, linear regression models were fitted separately for TSW and HLW. For TSW, simple regressions were performed with DI%, average seed weight, and pod index, followed by multiple regression models including DI% together with either average seed weight or pod index. For HLW, simple regressions were performed with pea height, average seed weight, and pod index, followed by multiple regression models including pea height together with either average seed weight or pod index. Model fit was evaluated using R^2^, adjusted R^2^, predicted R^2^, ANOVA, and residual diagnostics; variance inflation factors (VIFs) were used in multiple regression to assess multicollinearity. Correlation, PCA, and regression were used as complementary approaches to clarify why TSW and HLW showed different response patterns across cultivation treatments.

All univariate analyses, including GLM, ANCOVA/GLM, Tukey’s HSD, and linear regression, were performed in Minitab 17 (Trial version). The overview PCA based on pea yield, TSW, HLW, and DI% was performed in RStudio 2026.01.0+392 ‘’Apple Blossom’’ (R 4.5.2) and visualized using the ggplot2 package. The focused PCA, correlation heat map based on Spearman’s rank correlation coefficients, and additional graphical outputs, including the regression plots and LER figure, were prepared using Minitab 17 and Microsoft Excel 2019. Data visualization was therefore performed using a combination of Minitab 17, RStudio (R), and Microsoft Excel.

## 5. Conclusions

Under the low natural disease pressure recorded in this two-year field study, pea–cereal intercropping modified pea disease intensity, pea component performance, seed physical quality, and land-use efficiency in companion-species-dependent ways. However, this disease response should be interpreted within the low disease-pressure range recorded in this study because disease intensity remained low in both seasons and did not show a significant independent effect on pea grain yield, TSW, or HLW once the year and cultivation system were taken into account.

Pea component responses to intercropping were clearly companion-species-dependent. Pea grain yield was generally reduced under intercropping, but the magnitude of this reduction differed among cereal companions. Among the tested mixtures, pea–triticale provided the most balanced outcome, combining high land-use efficiency with pea yield comparable to the sole crop, whereas pea–oat showed the lowest DI% but also the lowest pea grain yield. These results indicate that reduced disease intensity did not automatically translate into better pea yield performance.

Additional association analyses showed that the two seed physical quality traits did not respond identically. TSW was more closely associated with seed-filling-related traits, whereas HLW was linked to a different structural dimension represented mainly by pea height. Thus, increases in TSW and HLW under intercropping should not be interpreted as equivalent responses or assumed to arise from the same biological processes.

Overall, the present study shows that pea–cereal intercropping can modify pea powdery mildew, pea performance, and land-use efficiency in a companion-species-dependent manner under temperate continental conditions. Further studies under wider disease gradients, including controlled or artificially increased infection, together with repeated disease assessments and direct measurements of canopy and belowground interactions, are needed to clarify the disease-management value of these systems and the mechanisms underlying pea yield–quality trade-offs.

## Figures and Tables

**Table 1:** Effects of year, cultivation system, and their interaction on pea yield, seed quality traits (TSW and HLW), and powdery mildew disease index (DI%) (*p*-values).

Factors	Yield	TSW	HLW	Powdery Mildew Disease Index
Year	0.002	0.005	<0.001	<0.001
Cultivation system	<0.001	<0.001	0.002	<0.001
Year x Cultivation system	0.194	0.266	0.225	0.001
DI% (covariate term in ANCOVA/GLM)	0.221	0.125	0.319	–

*p*-values for Year, Cultivation system, and Year × Cultivation system were obtained from GLM analyses for each response variable. The DI% row reports *p*-values for the DI% covariate term in separate ANCOVA/GLM models for yield, TSW, and HLW (with Year, Cultivation system, and their interaction included as factors). Dashes indicate that a term was not applicable to the corresponding response.

**Table 2:** Pea grain yield, thousand-seed weight (TSW), hectoliter weight (HLW), and powdery mildew disease index (DI%) in sole cropping and pea–cereal intercropping systems in 2018 and 2019 (mean ± SE).

Cultivation	Yield (t ha^−1^) 2018	Yield (t ha^−1^) 2019	TSW (g) 2018	TSW (g) 2019	HLW (kg hL^−1^) 2018	HLW (kg hL^−1^) 2019	PM (DI%) 2018	PM (DI%) 2019
Pea sole crop	1.145 ± 0.142	1.488 ± 0.108	102.6 ± 2.07	95.72 ±2.22	61.47 ± 0.56	64.96 ± 3.03	13.05 ± 0.14	14.87 ± 0.25
Intercrop with wheat	0.88 ± 0.06	1.057 ± 0.11	128.13 ± 1.50	127.25 ± 1.00	66.54 ± 1.47	72.73 ± 1.04	5.20 ± 0.194	6.70 ± 0.319
Intercrop with triticale	1.176 ± 0.043	1.488 ± 0.063	126.32 ± 1.68	125.10 ± 0.866	66.79 ± 0.803	73.85 ± 0.948	5.025 ± 0.077	5.725 ± 0.159
Intercrop with rye	0.898 ± 0.036	0.882 ± 0.107	129.78 ± 0.602	127.07 ± 1.73	66.25 ± 1.87	68.65 ± 3.37	4.25 ± 0.201	5.12 ± 0.072
Intercrop with oat	0.524 ± 0.067	0.768 ± 0.070	129.15 ± 0.965	126.90 ± 0.853	64.65 ± 1.52	75.00 ± 1.08	3.987 ± 0.362	3.70 ± 0.247

Values are means ± SE (*n* = 4) for each cultivation treatment within each growing season. Table 2 presents year-specific treatment means, whereas pooled inferential comparisons across years for yield, TSW, and HLW are presented later in Section 2.2 because the Year × Cultivation system interaction was not significant for those traits.

**Table 3:** Powdery mildew disease index (DI%) of pea in sole cropping and pea–cereal intercropping combinations in 2018 and 2019 (mean ± SE). Different letters within a column indicate significant differences among cultivation treatments (Tukey’s HSD, *p* ≤ 0.05).

Cultivation	DI% 2018 (Mean ± SE)	DI% 2019 (Mean ± SE)
Sole-cropped pea	13.05 ± 0.14 ^B^	14.87 ± 0.25 ^A^
Intercrop with wheat	5.20 ± 0.19 ^DE^	6.70 ± 0.32 ^C^
Intercrop with triticale	5.03 ± 0.08 ^DEF^	5.73 ± 0.16 ^CD^
Intercrop with rye	4.25 ± 0.20 ^EFG^	5.12 ± 0.07 ^DE^
Intercrop with oat	3.99 ± 0.36 ^FG^	3.70 ± 0.25 ^G^

Means sharing at least one letter are not significantly different (Tukey’s HSD, *p* ≤ 0.05).

**Table 4:** Tukey’s HSD groupings (pooled across years) for pea grain yield, thousand-seed weight (TSW), and hectoliter weight (HLW) under sole cropping and pea–cereal intercropping combinations. Pooled comparisons are presented because the Year × Cultivation system interaction was not significant for yield, TSW, and HLW.

Cultivation	Yield (t ha^−1^)	TSW (g)	HLW (kg hL^−1^)
Sole-cropped pea	1.3165 ^A^	99.162 ^B^	63.218 ^B^
Intercrop with wheat	0.9685 ^B^	127.687 ^A^	69.635 ^A^
Intercrop with triticale	1.3319 ^A^	125.712 ^A^	70.322 ^A^
Intercrop with rye	0.8904 ^BC^	128.425 ^A^	67.448 ^AB^
Intercrop with oat	0.6463 ^C^	128.025 ^A^	69.823 ^A^

Within each trait (column), means sharing at least one letter are not significantly different (Tukey’s HSD, *p* ≤ 0.05).

**Table 5:** Total intercrop yield (pea + cereal component yield), component yields, and component shares in pea–cereal intercropping combinations during the 2018 and 2019 growing seasons. Values are presented as descriptive system-level means ± SE (*n* = 4).

Season	Intercrop	Total Intercrop Yield (t ha^−1^)	Pea Component (t ha^−1^)	Cereal Component (t ha^−1^)	Pea Share (%)	Cereal Share (%)
2018	Pea–oat	4.102 ± 0.233	0.524 ± 0.065	3.578 ± 0.183	12.7 ± 1.0	87.3 ± 1.0
2018	Pea–wheat	5.629 ± 0.157	0.880 ± 0.060	4.749 ± 0.201	15.7 ± 1.3	84.3 ± 1.3
2018	Pea–rye	6.270 ± 0.166	0.898 ± 0.036	5.372 ± 0.210	14.4 ± 0.9	85.6 ± 0.9
2018	Pea–triticale	5.746 ± 0.118	1.176 ± 0.043	4.570 ± 0.096	20.5 ± 0.4	79.5 ± 0.4
2019	Pea–oat	5.059 ± 0.345	0.768 ± 0.071	4.290 ± 0.361	15.3 ± 1.6	84.7 ± 1.6
2019	Pea–wheat	5.312 ± 0.275	1.057 ± 0.110	4.255 ± 0.186	19.7 ± 1.1	80.3 ± 1.1
2019	Pea–rye	6.047 ± 0.279	0.882 ± 0.107	5.165 ± 0.214	14.5 ± 1.3	85.5 ± 1.3
2019	Pea–triticale	4.665 ± 0.244	1.488 ± 0.108	3.176 ± 0.213	32.0 ± 1.2	68.0 ± 1.2

**Table 6:** Ordinal 0–9 scale used for scoring pea powdery mildew severity (adapted from Saari and Prescott [64]).

Value	0	1	2	3	4	5	6	7	8	9
Degree of leaf infection	Without infection	0.1–5%	5.1–10%	10.1–17%	17.1–25%	25.1–50%	50.1–75%	75.1–90%	90.1–95%	≥95%

## Data Availability

Data are reported within the article and Supplementary Material.

## Supplementary Materials

The following supporting information can be downloaded at: https://www.mdpi.com/article/10.3390/plants15101437/s1. Figure S1: Complementary exploratory regression analyses—Plot-level linear relationships between (a) thousand-seed weight (TSW) and powdery mildew disease index (DI%) and (b) hectoliter weight (HLW) and pea height. Lines represent fitted linear regressions; shaded areas indicate 95% confidence intervals.; Figure S2: Principal component analysis (PCA) biplot summarizing pea grain yield, thousand-seed weight (TSW), hectoliter weight (HLW), and powdery mildew disease index (DI%) across cultivation treatments in 2018 and 2019; points represent plot-level observations and arrows represent variable loadings.; Table S1: Partial land equivalent ratios for pea (pLER) and cereal (cLER) and total yield-based land equivalent ratio (LER) in pea–cereal intercropping combinations during the 2018 and 2019 growing seasons. Values are mean ± SE (*n* = 4). Total LER was calculated as pLER + cLER.; Table S2: Post-winter established plant density (plants m−2) of pea and cereal components in sole crops and intercrops in 2018 and 2019 (mean ± SE, *n* = 4).; Table S3: Long-term monthly climatic normals (1991–2020) for the Rimski Šančevi meteorological station, Novi Sad, Serbia (45°20′ N, 19°51′ E; 84 m a.s.l.).
