# Supplementary material for: Pea Powdery Mildew and Pea Performance in Pea–Cereal Intercropping Under Temperate Continental Field Conditions: Yield, Seed Physical Quality, and Land-Use Efficiency Under Low Natural Disease Pressure
Source: Plants (Basel). 2026 May 8;15(10):1437. doi: 10.3390/plants15101437 (PMC13210937; doi:10.3390/plants15101437)
Supplement: Supplementary file 1 [file plants-15-01437-s001.zip › plants-4273194-supplementary.pdf]

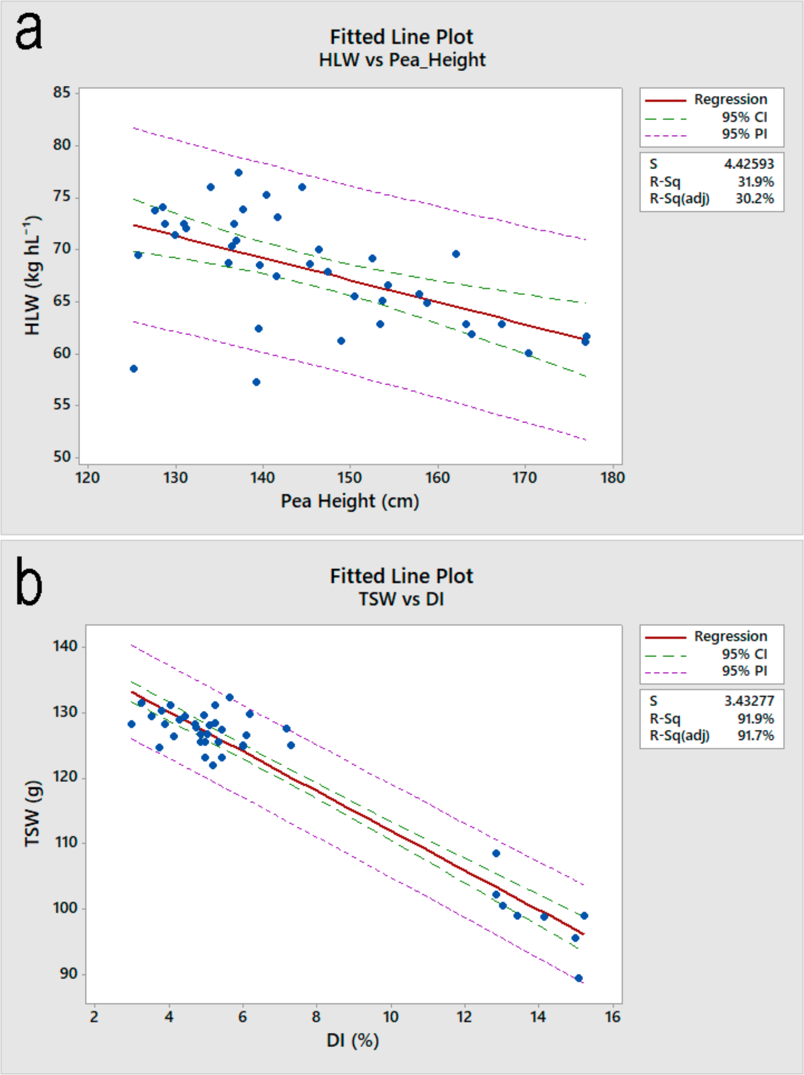

Figure S1. Complementary exploratory regression analyses - Plot-level linear relationships between (a) thousand-seed weight (TSW) and powdery mildew disease index (DI%) and (b) hectoliter weight (HLW) and pea height. Lines represent fitted linear regressions; shaded areas indicate 95% confidence intervals.

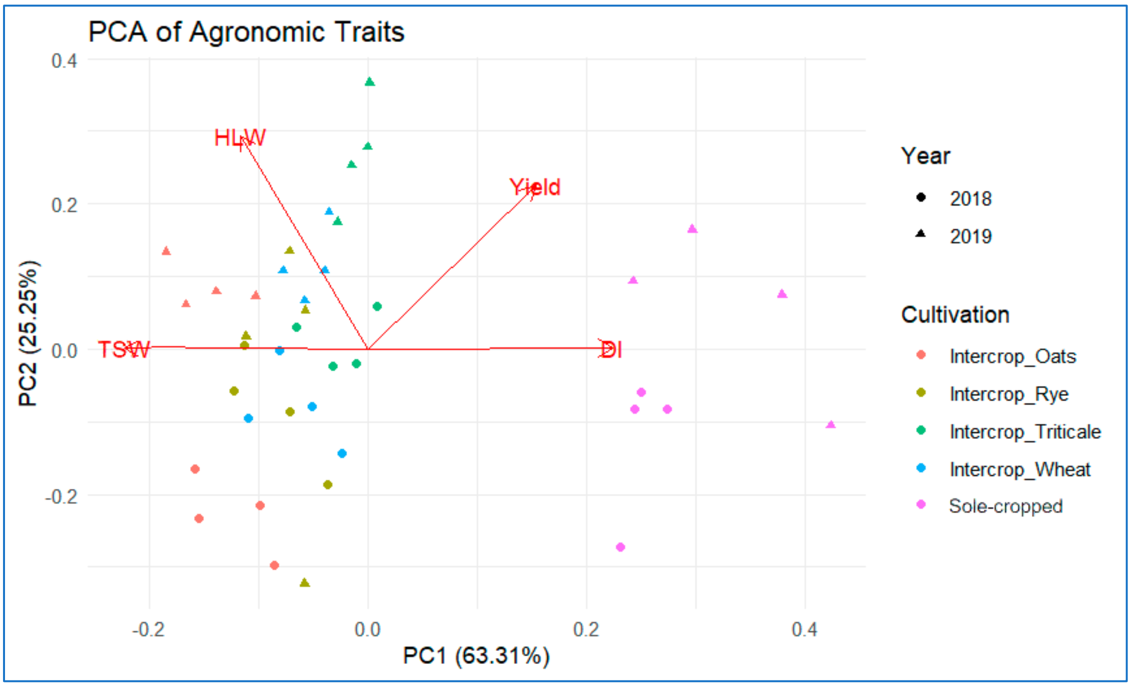

Figure S2. Principal component analysis (PCA) biplot summarizing pea grain yield, thousand-seed weight (TSW), hectoliter weight (HLW), and powdery mildew disease index (DI%) across cultivation treatments in 2018 and 2019; points represent plot-level observations and arrows represent variable loadings.

Table S1. Partial land equivalent ratios for pea (pLER) and cereal (cLER) and total yield-based land equivalent ratio (LER) in pea–cereal intercropping combinations during the 2018 and 2019 growing seasons. Values are presented as descriptive system-level means ± SE (n = 4). Total LER was calculated as pLER + cLER.

Season	Intercrop	pLER (mean ± SE)	cLER (mean ± SE)	LER (mean ± SE)
**2018**	Pea–oat	0.458 ± 0.058	0.568 ± 0.029	1.026 ± 0.083
**2018**	Pea–wheat	0.769 ± 0.052	0.621 ± 0.026	1.390 ± 0.043
**2018**	Pea–rye	0.785 ± 0.031	0.649 ± 0.025	1.434 ± 0.021
**2018**	Pea–triticale	1.027 ± 0.038	0.584 ± 0.012	1.611 ± 0.048
**2019**	Pea–oat	0.516 ± 0.047	0.543 ± 0.046	1.059 ± 0.069
**2019**	Pea–wheat	0.710 ± 0.074	0.578 ± 0.025	1.288 ± 0.097
**2019**	Pea–rye	0.593 ± 0.072	0.742 ± 0.031	1.335 ± 0.091
**2019**	Pea–triticale	1.000 ± 0.043	0.499 ± 0.033	1.498 ± 0.065

Table S2. Post-winter established plant density (plants m^{-2}) of pea and cereal components in sole crops and intercrops in 2018 and 2019 (mean ± SE, n = 4).

Cultivation system	2018 pea component	2018 cereal component	2019 pea component	2019 cereal component
Sole-cropped pea	67.75 ± 3.99	—	72.50 ± 5.20	—
Pea + wheat	44.25 ± 3.73	129.75 ± 7.13	47.50 ± 4.33	141.00 ± 7.72
Pea + triticale	37.75 ± 1.97	108.25 ± 2.06	41.25 ± 2.39	117.00 ± 1.91
Pea + rye	40.00 ± 1.22	102.75 ± 4.71	42.50 ± 1.44	111.00 ± 5.51
Pea + oat	45.50 ± 0.87	112.50 ± 3.71	49.25 ± 1.49	121.25 ± 4.11
Sole-cropped wheat	—	261.25 ± 11.95	—	281.00 ± 13.20
Sole-cropped triticale	—	200.50 ± 8.15	—	217.00 ± 9.15
Sole-cropped rye	—	158.25 ± 6.84	—	170.00 ± 7.39
Sole-cropped oat	—	166.75 ± 19.18	—	180.00 ± 21.10

Post-winter plant density was assessed in late March (30 March 2018 and 29 March 2019) within 1 m^2 per replicate.

Table S3. Long-term monthly climatic normals (1991–2020) for the Rimski Šančevi meteorological station, Novi Sad, Serbia (45°20′ N, 19°51′ E; 84 m a.s.l.).

Variable	Jan	Feb	Mar	Apr	May	Jun	Jul	Aug	Sep	Oct	Nov	Dec	Annual
Mean air temperature (°C)	0.7	2.3	7.0	12.4	17.3	20.9	22.5	22.4	17.2	12.0	6.8	1.8	11.9
Relative humidity (%)	85.5	80.2	70.8	66.4	67.9	69.7	68.2	67.4	72.5	77.1	82.1	86.7	74.5
Precipitation (mm)	38.9	36.4	38.6	46.6	77.3	92.2	68.1	59.7	58.8	58.6	51.5	49.1	675.8

Source: Republic Hydrometeorological Service of Serbia (RHMZ), standard climatological period 1991–2020.
